# Two Obstacles in Response Efforts to the Ebola Epidemic in the Provinces of North Kivu and Ituri in the Democratic Republic of the Congo: Denial of and Rumors about the Disease

**DOI:** 10.29245/2578-3009/2023/S3.1104

**Published:** 2023-05-12

**Authors:** Nkechi G. Onyeneho, Ngozi Idemili Aronu, Ijeoma Igwe, Joseph Okeibunor, Tieman Diarra, Amadou Baïlo DIALLO, Bairo Hamadou, Barry Rodrigue, Mamoudou Harouna Djingarey, Zabulon Yoti, N’da Konan Michel Yao, Ibrahima Socé FALL, Dick Chamla, Abdou Salam Gueye

**Affiliations:** 1 University of Nigeria, Nsukka; 2 World Health Organization, Switzerland; 3 Independent Consultant, Mali; 4 Independent Public Health Expert, Niger

**Keywords:** Denial, Rumors, Ebola, Vaccine, Ebola treatment center, Hospital, Safe and dignified burial, Reluctance, Resistance, Violence

## Abstract

Denial and rumors are two major obstacles impairing the implementation of activities in response to the Ebola virus disease (EVD) epidemic. This study investigated the roles of denial and rumors, among other challenges, in complicating the response to the EVD outbreak in the North Kivu and Ituri provinces of the Democratic Republic of the Congo. A total of 800 randomly selected respondents were surveyed using a structured questionnaire. In-depth interviews were conducted with 17 community religious and opinion leaders, as well as Ebola survivors. Furthermore, 20 focus group discussions were conducted with adult and youth male and female participants, and health care workers. The results revealed that the existence of the disease is widely denied by many, including political leaders, village chiefs, neighborhood chiefs, street chiefs, avenue chiefs, and members of the general population. These individuals generally consider the EVD to be the result of a misbehavior or a curse; consequently, the general population, including community members, teachers, and even health care professionals, refuse to comply with the authorities’ strategies to fight the epidemic. Rumors are another obstacle in response efforts. Rumors pertaining to the denial of the existence of the EVD, as well as the epidemic, Ebola treatment centers, hospitals, vaccines, and safe and dignified burials have been identified. Rumors about the EVD and the response, spread by clerics, traditional therapists, men, and women, including healthcare professionals in focus group discussions, portrayed the EVD as an invention, as if the virus had been created. The response to the EVD has been marked by these two constraints, which have often hindered the involvement of community members in the fight against the disease.

## Introduction

National authorities in the Democratic Republic of the Congo (DRC) led the response to the Ebola outbreak, partnering with the numerous communities. However, community involvement faced two major challenges, namely, denial of and rumors about the Ebola virus disease (EVD) and the epidemic, which have hindered the implementation of response activities.

Various groups of people have denied the existence of the EVD, including some of the most influential people in the communities, such as political leaders, village chiefs, neighborhood chiefs, street chiefs, and avenue chiefs. They attribute the disease to misbehavior, a taboo, or to a curse, and thus refuse to adhere to the strategies implemented by authorities to fight the epidemic^1^. Furthermore, other members of the community such as the public, teachers, and even healthcare professionals have denied the existence of the disease. This situation has been particularly harmful to healthcare workers, who are often infected by the EVD due to their disbelief in the existence of the disease and consequently not taking the necessary measures to prevent contamination in their professional practice^2^.

Communities face several challenges in the face of this deadly disease. Denial by community members was one of the biggest obstacles to response efforts^3,4^. It caused reluctance, indifference, and refusal to participate in the response activities among community leaders and members. It has also led to acts of violence against members of the response teams. Denial of the existence of the EVD has been expressed by various groups of individuals, including both men and women, as well as older adults and the youth. Among these individuals, the youth are often the ones initiating acts of violence against the response teams.

Another challenge faced in EVD response include the rumors about the reality of the EVD, the epidemic, the treatment centers, the hospitals, the vaccine, and safe and dignified burials (SDB). These rumors are spread by clerics, traditional healers, men, women, and even healthcare professionals. These rumors portray the EVD as an invention, as if the virus had been created^5^. Moreover, they relate the disease to witchcraft or an evil spell. Other rumors consider it as a scheme, as the business of a white man, concocted to generate profits and exterminate populations, or disrupt elections. Some individuals doubt the existence of the disease, because according to them, the sick individuals are not visible to the public. Rumors about the vaccine discourage public support for immunization, as vaccines are depicted as a tool to reduce the life expectancy of the population, to inhibit reproduction, to spread infections, and to weaken intelligence. Likewise, rumors that suggest the existence of real and fake vaccines do not reassure the population, who believe real vaccines are only administered to healthcare workers.

Ebola Treatment Centers (ETCs) and hospitals are rumored to be places for organ removal, genital mutilation, or inducing death, leading people to fear these health-care facilities. While some patients refuse to visit these places, others flee after being admitted. SDBs are also the subject of rumors. Consequently, community members have begun to refuse having these rituals. Some rumors mention that the number on a person’s body bag indicates the number of people who will die in the deceased person’s family.

Denying the existence of the EVD and the associated rumors have hampered the implementation of response activities to fight the epidemic in the provinces of North Kivu and Ituri in the DRC. In fact, they have obstructed the implementation of response activities in the entire country.

This study focuses on the denial of the existence of the EVD and the rumors regarding the disease and the epidemic, and examines the instruments to fight it.

## Study Design and Methods

### Study Design

This study was designed to explore and document experiences and lessons around the response to the 10th EVD outbreak in the North Kivu and Ituri provinces of the DRC. A cross-sectional design and mixed methods technique of data collection were considered. The design allowed multiple windows of data collection. The mixed methods approach brought the benefits of both quantitative and qualitative research techniques and guaranteed the integrity, robust interpretations, and conclusions that this type of evaluation required.

#### Selection of Study Area and Population

The study was carried out in the North Kivu and Ituri provinces where the 10th EVD outbreak occurred in the DRC.

**Ituri** is one of the 26 provinces of the DRC. Its capital is the city of Bunia. The Ituri Rainforest is found in this area. It is located northeast of the Ituri River and on the western side of Lake Albert. Ituri is a high plateau region (2000–5000 m) that has a large tropical forest but also the savannah landscape. The district has rare fauna, including the okapi, the national animal of the Congo. An important species of flora is found here—the Mangongo, the leaves of which are used by the Mbuti to build their homes. The population is composed primarily of Alur, Hema, Lendu, Ngiti, Bira, and Ndo-Okebo, with differing figures on the group constituting the largest percentage of the population in the province. The Mbuti, a pygmy ethnic group, reside primarily in the Ituri forest near the Okapi Wildlife Reserve, although some Mbuti have been forced into urban areas by deforestation, over-hunting, and violence. The Kilo-Moto gold mines are partly located in Ituri. In the beginning of the 21st century, petroleum reserves were discovered by Heritage Oil and Tullow Oil on the shores of Lake Albert.

**North Kivu** (French: *Nord-Kivu*) is a province bordering Lake Kivu in eastern DRC. Its capital is Goma. North Kivu borders the provinces of Ituri to the north, Tshopo to the northwest, Maniema to the southwest, and South Kivu to the south. To the east, it borders the countries of Uganda and Rwanda. The province consists of three cities—Goma, Butembo, and Beni—and six territories—Beni, Lubero, Masisi, Rutshuru, Nyiragongo, and Walikale. The province is home to the Virunga National Park, a World Heritage Site home to endangered mountain gorillas. Except for the heightened insecurity and isolation due to rebel activities, North Kivu shares similar demographics with Ituri. The province is politically unstable, and has been a flashpoint of military conflicts in the region since 1998.

The **2018 or 10th Kivu Ebola outbreak** began on August 1, 2018, when it was confirmed that four individuals had tested positive for the Ebola virus in the eastern region of Kivu in the DRC. The Kivu outbreak included the Ituri Province, after the first case was confirmed on August 13^6^. This outbreak started just days after the end of the 2018 Équateur province DRC Ebola virus outbreak^5^.

The affected province and general area are currently undergoing a military conflict, which is hindering treatment and prevention efforts. The WHO Deputy Director-General for Emergency Preparedness and Response has described the combination of military conflict and civilian distress as a potential “perfect storm” that could lead to a rapid worsening of the outbreak^2^. Due to the deteriorating situation in North Kivu and surrounding areas, on September 27, the WHO raised the risk assessment at the national and regional level from “high” to “very high”^2^.

The study population comprised adults aged ≥18 years living in the community, as well as the response team members. In 2010, the estimated population of North Kivu was 5,767,945. With an annual growth rate of 3.2%, the population in 2019 was estimated to be 7,658,406 and 5,360,884 for the general and adult populations, respectively. In 2005, the estimated population of Ituri was 4,037,561. Hence, the 2019 population was estimated to be 6,275,305 and 4,392,714 for the general and adult populations, respectively.

The response team consisted of over 10,000 people in different response pillars, including surveillance, risk communication, social anthropology, and vaccination. Others included infection prevention and control, treatment and care, and safe and dignified burial, as well as security, logistics, and administration.

#### Sample Size Estimation and Sampling Strategy

This was an exploratory study in which a sample of the study population was taken. With an assumed 50% chance of accepting Ebola intervention control interventions at a confidence interval of 95%, and with an error margin of 5%, a sample size of 384 participants in each province was computed as necessary for the quantitative study. This was rounded up to 800 across both provinces to allow for losses. The final size of the sample depended on data saturation after an initial pair was collected from each category of respondents.

A multi-stage sampling technique was adopted to select the communities, households, and respondents in this study. Two administrative areas (the epicenters of the EVD outbreak in each province) were purposively selected. Ten communities were then randomly selected from each of the two administrative areas in the province.

The center of the selected community was the reference point where the team spun a pencil to determine the first route and household, and thereafter moved to the right to pick the next household; this was continued until the number of households to be sampled was satisfied. Where there was a *cul-de-sac*, the team retraced their steps, and a turn to the left and then to the right was made to continue the sampling process.

Once in a selected household, an adult (≥ 18 years) was randomly selected for inclusion as a participant in the study. The sex of the participants was carefully alternated; if a male participant was selected in household number one, in the next household, the focus was on selecting a female participant.

### Methods

The study was conducted using a mixed methods approach, with data collection involving in-depth interviews (IDIs), focus group discussions (FGDs), and surveys using structured questionnaires. This type of study requires a strong focus on individual actors rather than state actors^7^.

### Techniques of Data Collection

The community leaders include traditional, political, religious and opinion leaders

A set of questions covering different thematic areas were developed to guide the discussions. The questions gathered information on health care services in the community, awareness of, and practices related to the EVD, as well as an assessment of the different pillars of the response interventions.

For the FGDs, 8−12 persons were selected for each session. A minimum of two FGDs were conducted in the selected communities. Separate FGDs were conducted for male and female participants in each of the communities. A total of eight FGDs were conducted in each province.

IDIs were conducted in each community where FGDs were carried out. The IDIs were held with community or opinion leaders of the selected communities and team leaders of the response pillars. Interviews were used to explore people’s opinions, views, and attitudes to understand practices related to the outbreak and response efforts, and other socio-cultural factors that may have influenced people’s attitudes toward disease response. The FGD guide was used for the IDI, focusing on the thematic areas of interest to the evaluation.

A structured questionnaire was used for collecting quantitative data from households. The questionnaire addressed all the indicators that were used for answering the research questions. The questionnaire was structured with results from the qualitative study. It was categorized into the following sections: socio-demographic data, perception of health problems in the community, knowledge of the EVD, perceived epidemiology of Ebola in the communities, and sources of information on Ebola. Others included issues on communication and community engagement, infection prevention and control in the communities, vaccination, surveillance, and treatment and care. Further, some questions covered themes of EVD, psychosocial issues, and logistics and security issues.

All interviews and discussions were tape-recorded, and detailed notes were taken simultaneously, including verbal citations. Tape-recorded interviews were transcribed according to standard rules. Observations were also recorded, and together with data from the discussions and interviews, they were triangulated with the quantitative data to arrive at the study findings and conclusions.

#### Training and Pilot Trials

All instruments were ***translated*** into Swahili and French, the common languages spoken in the communities, and back-translated to English for clarity of meaning. In each province, ***10 research assistants***, with substantial experience in community interactive research, and the use of qualitative and quantitative techniques and cultural sensibility, were recruited and trained for three days in Beni, and another three days in Bunia on the study objectives, and the use of the instrument for data collection. Training also included data entry into Atlas.ti template (qualitative data) and Epi Info (quantitative data). The instruments were reviewed after training for clarity, understanding, and sensitivity. Each province had a ***supervisor*** who worked with the principal investigator on data quality monitoring, safety advisory, and ethical conduct of the research, including the management of informed consent procedures. The study was conducted first in Ituri, then in North Kivu. The lessons learned from Ituri were used to manage the process in North Kivu, a province with more security and logistics challenges. The ***data analyst*** developed and pre-tested the template for data entry and analysis using the pilot test output. Fieldwork took approximately 20 days to complete in each province, prior to data analysis and report writing.

### Data Management

All **quantitative data** were double-checked by the researcher before being entered into a computer. Data were entered into Epi Info and processed using SPSS. Descriptive statistics were used to determine proportions of various categories of respondents and indicators, and for data comparison. Frequency tables and graphic illustrations were used for presenting the data.

**Qualitative data** consisting of FGDs and IDIs were transcribed from audio records to text. All textual data were analyzed using the Atlas.ti software package. Data were analyzed according to themes corresponding to the indicators in the quantitative data and triangulated during presentation to enable complementary and analogous interpretation.

Given the continuous analytical process involved in qualitative analysis, it is important to note that the initial analysis of the key informant interviews and FGDs informed the final development of the structured questionnaire to be used in the study. This further enhanced triangulation between the two sets of data to be collected. While the quantitative results provided us with statistical conclusions, the qualitative results placed emphasis on what was said and provided illustrative quotes that gave context and depth to the quantitative results.

### Ethical Considerations

The principle of do-no-harm was adhered to in the study. Informed study approval was obtained at the levels of the province, local administration, community, and household, where informed consent was obtained from all individuals that were involved in the study. The WHO/AFRO Ethics Review Committee provided ethical approval for the study. All researchers attended the mandatory training, which included substantial discussions on ethical issues in research. Fifty percent of the research assistants were women, to facilitate same-sex interviews and moderation of FGDs. The assistants were also trained and mandated to comply with child protection and gender sensitivity protocols in the process of data collection and during visits.

## Results

Members of the community including high ranking individuals, deny the existence of the disease. Neighborhood leaders either question the existence of the EVD themselves or quote community members who do not believe it exists. Some of the comments about the disease cross territories. For example, the deputy mayor of Oicha spoke of politicians in Butembo, who denied the existence of the EVD. He thought these comments were unfortunate, especially because he believed that politicians should rather help the population to understand the reality of the disease and take action to fight it.

The chief of the Baye district in Butembo lamented this situation in the following terms: *“Politicians have destroyed the memory of the inhabitants of Butembo. While information was given on the ways to prevent it, the politicians gave a contrary message, saying that the disease does not exist. This has pushed the population into resistance.”* This statement was also cited by an employee of the Butembo town hall, who criticized the fact that the politicians did not intervene to contradict themselves and reassure the population. He found it even more regrettable that the very person who spread hostile messages about the *response* had later changed his attitude and decided to get vaccinated.

A traditional healer in Butembo stated that the population did not believe in the existence of the EVD. In these circumstances, messages from elected officials denying the existence of the disease were not likely to help in the implementation of the *response* activities to fight the epidemic. The traditional therapist said: *“People believe that Ebola does not exist. For them, it is a disease created by the State to eliminate the Nande people. It is there, people are dying, and that is when the disease comes. That is why people did not believe in this disease. Everything appears to be another mission to eliminate the Nande people*.” Here, we realize the full weight of the rumors from the people’s perception of the disease, and will discuss these rumors ahead.

The village chief of Kyamase in the Kalengehya health zone, also denied the existence of the EVD. He even talked about people’s loss of culture due to the interventions during the Ebola outbreak. He shared: *“Ebola does not exist. We have grown up not listening to all your lies. We had our only doctor that you corrupted. He told us about the Ebola virus. I grew up suffering from hemorrhage. All these symptoms they tell us about are lies because they are our usual diseases. We are now suffering from malaria and from poisoning. We have lost our whole culture because of you.”*

There is often a mix of information emerging from the *response* efforts and from the rumors that are circulating among the population. He continued: *“As we hear you talk about the disease, there is not only a certain group of people who are forced to receive this disease! We are all concerned and especially the doctors who are really exposed, but they do not die. We concluded that our women, our children, our daughters, our family members of blood group O are the most targeted.”*

This conclusion is based on a rumor that is present in many forms within the communities. He stated that everyone is affected by the disease, and that he believes that people from a particular blood group are targeted. They are not exposed to it as the doctors said, rather they are targeted.

When village chiefs spread rumors, one can understand the extent to which they reach, as these leaders have a strong influence over many members of the community. Information is received and processed selectively. Access to information does not always change one’s attitude toward the disease, because it is often considered a lie. He stated: *“People have been informed about the Ebola here, by the health people. But we see that these people, who come to our homes, always lie.”* This attitude makes it challenging to provide information since it is constantly analyzed and compared to what has already been said. Everything appears as though it will not make a difference, because in the end, it is denial that prevails.

The village chief was not taking any action to engage his community in the fight against the EVD. He stated: *“Here in Kyamase, I am not doing anything about the Ebola virus because I don’t believe in your disease, I am just observing what you and your doctors are doing.”* When asked about the role of the community in providing information on the EVD, he considered it difficult to answer because, according to him, the people have no information on the disease. As for the prevention measures taught to avoid infection, such as washing hands, he believed it is in the nature of things. He said, *“We don’t even have a clue about the disease, how can you expect us to get involved in your private affairs! If we wash both hands when we travel, it doesn’t mean that we grew up without knowing that we have to wash our whole body every time.”* Usually, the denial of the Ebola virus is not an obstacle to act on for the response in the fight against the epidemic. Community leaders do, however, play their institutional role, which provides an opportunity for interaction with members of the response teams. They might even express their expectations from the response, which they see as an opportunity to ask for something in return. The following comments from the same leader illustrate this.

Despite all his opposition to the *response* activities and his denial of the EVD, this neighborhood chief was still taking positive action to fight the disease: *“I do everything to inform the members of my community of all those who arrive at my house. There are members of the community who listen to us and others who [do not], who resist.”* He suggested the following to protect the members of his community from the virus: *“You should give us clean water first. We ask you to come and inform people before vaccinating them, to also provide us with mosquito nets, and to check that the prevention measures are put into practice.”*

In this chief’s village, there were traditional tools for disseminating information, which he called *Kibuyi*. However, some advice is followed despite the refutation of the existence of the EVD: *“We wash both hands; we no longer eat our animals that die of natural causes. We no longer go to the funeral site without the right information from you. But these situations have led to breaking the relationships we have with each other.”* Nonetheless, he ended up talking about the responsibility of the *response* personnel and the community. He stated that information from the *response* staff should be a recommendation to community members on how to take action to protect themselves.

The village chief of Kyamase was not the only person to question the existence of the EVD and the legitimacy of the *response*. When a leader, who is a potential source of influence, does not endorse something, he will not get others in his community to do the opposite. There are people all over who do not believe in the reality of the Ebola virus. The Pasisi neighborhood chief in the Boikene health area claimed that the incidents that occurred during the Ebola *response* activities were due to misunderstandings. He said: *“People think the disease is fake, that it does not exist*.”

The denial of the disease was expressed in communities in the provinces of North Kivu and Ituri. The head of the Kilomoto avenue in the Ngezi district of Bunia deplored the fact that the population that has information does not always share it with other community members. He stated that people claim the EVD does not exist and that it is a business. The chief of the Bukavu avenue in Butembo corresponded with this statement.

Some people deny the existence of the EVD, because they are unaware of any cases in their village. The chief of the Kandate village in the Mandima health area shared: *“We will believe in the Ebola virus the day we see it ourselves, in our village. Then, we will call the members of the* response *[team] ourselves, so they can come and intervene. For now, we know that the Ebola virus does not exist. A girl from a neighboring village died in our health center here. We, from the village, know that the girl was suffering from* ***kapuru***, *but your new doctors who came to exterminate us for the sake of money declared that it was Ebola and that pushed us to destroy the health structure that you will see there.”*

However, not everyone denied the existence of the disease. The chief of the Matembo district in the Boikene health area stated that there are two categories of people in his community: those who believe the Ebola virus exists and those who believe it does not. Rumors have led to the denial of the EVD. A nurse at the Makasi health center said: *“When the epidemic was taking hold, there were a lot of rumors. Some claimed it was a politicized disease. Others, that the Ebola is a business, and that people are there to get money, but that the disease is fake.”*

The nurse in charge of the Makasi spoke about the difficulties that healthcare workers face regarding the population. He shared the following: *“The first struggle we face during our work, besides the risk of contamination, is the misunderstanding between health workers and the population. The community members say that the health workers are getting paid to say that the disease exists. The community members argue that the disease does not exist*.”

Throughout our interviews with village chiefs, avenue chiefs, and street chiefs, a few of them claimed to not believe in the existence of the EVD. However, most of them do believe the virus exists and are contributing to the fight against the Ebola virus, as discussed below. During the FGDs, we met many individuals who did not believe in the EVD. In some cases, discussions were held between those who had opposing opinions on the reality of the Ebola virus.

In all the FGDs, participants stated that they knew members of their community, who did not believe in the existence of the Ebola virus. Likewise, some revealed to be among those who do not believe in the existence of the Ebola virus themselves. During a discussion with men in Kalengehya, one participant stated: *“There are those who say Ebola does not exist. They are destroying the real information about Ebola.”*

In the Mbimbi neighborhood in the Mabasele health area, during an FGD, a man explained the reasons why members of his community have not been vaccinated. He mentioned: *“We know that Ebola does not exist here. That is why we have not been vaccinated.”* In a discussion in Pasisi, in the Boikene health area, a participant claimed that the young are the ones who deny the existence of the EVD the most. Some used the denial of the disease to act against other members of their community. A participant in an FGD in the Bukavu avenue in Butembo stated: *“I resisted, together with a friend. Even where people were mourning, we would go and disturb, saying that the Ebola does not exist. Then, my friend felt sick and called the nurses of Itave himself. They said the person we buried was a confirmed case. My friend spent seven days in the Itave hospital, and I always went to visit him. After seven days, fortunately, he was discharged and until now I cannot confirm whether the disease exists or not. I don’t know the symptoms of the Ebola virus.”* The same person said that in the neighborhood, there are no preventive measures, and that it is not necessary for him to take precautions for a disease that does not exist.

Some people denied the existence of the disease, because they had not seen a case. In a discussion in Kavale, in the Kyondo health area, a woman shared: *“I have never seen a person with Ebola. If the health workers notice a spot in my eye, they will say it is Ebola and if I go for treatment, I will leave the hospital dead. So, I say that Ebola does not exist. Even when we go to the hospital because of a toothache, one of our old diseases that used to be treated well, they will say it’s Ebola. Even if a person who hangs himself, they will say it was Ebola, even malaria is said to be Ebola, so I don’t know if this disease exists. I know it is killing because I have never seen a person cured of Ebola. Even a person who comes to the hospital with a headache is being held for Ebola.”* Arguments were made to support the denial of the existence of the disease. In the same discussion, similar opinions were expressed. Some participants stated that they did not understand why animals did not die as much. Others shared anecdotes of people who would have fallen sick if the disease was a reality. These stories were motivated by rumors.

Even after receiving training on Ebola, it did not help to convince them of the reality of the disease. A woman in the same group said: *“I had been trained outside this village. We were told to always wash our hands with soap. I don’t like to be forced to do something and as I know that Ebola doesn’t exist, I couldn’t be forced to do it. In my opinion, I could not follow this advice because I know that Ebola does not exist.”* But not all the women questioned the existence of Ebola during the discussions. One woman shared: *“You know, there are always those who understand and those who don’t. People don’t understand at the same time. They don’t agree because many think that maybe the disease doesn’t exist. Also, others think that we don’t bury people and that the coffins are empty.”*

In a discussion in Boikene, in Beni, a man stated that everyone has their own way of receiving information and handling it. According to him, some people still consider that the EVD does not exist. Others think the Ebola has become a business, which is why the disease never ends. In a discussion in the Bukavu avenue in Butembo, a woman said: *“Some people who have been cured are seen as if they had been corrupted by the* response *to testify about a disease that does not exist.”* Another woman in a discussion in Kasinga, in the health area of Kalengehia, stated that the disease does not exist and that it is a set-up.

In a discussion in Kyamasi, two participants stated that the EVD is a business, and that it does not exist. In a discussion in Kalengehya, a man stated that he thought that many people did not believe in the existence of the EVD. He said: *“Everyone here has their own idea because most people say the Ebola does not exist. Even the relays have become enemies for some. That is why even the chiefs cannot hold meetings on this disease except when their conscience tells them to believe. It is only then that they will tell their families to apply rules of hygiene.”*

Visiting those who have recovered is an opportunity for many to monitor information or rumors about the EVD since they do not believe it. A member of a youth association mentioned: *“We have a religious leader who declares in his preaching that the Ebola is not a reality. He states it is a political set-up. The faithful come and say the same thing in the neighborhoods. They spread the words of the pastor.”* However, some people changed their minds and ended up implementing preventive measures. A man from the Bakumu avenue in the Ngezi district of Bunia said: *“I used to ignore preventive measures because I was not convinced that there was a risk of contracting Ebola. I was one of the people who said that Ebola does not exist.”*

During a discussion in the Mabolio neighborhood of Beni, a participant argued that no one should doubt the existence of the disease. She said: *“There is one of us whose child was cured after her treatment at the ETC. But I lost two daughters. My neighbor lost her father. I know many people who say that the disease is politics. But there have been cases. We’ve lost parents. It is a reality.”* In the same neighborhood, however, during another FGD with women, a participant shared: “*I don’t believe in this disease. I have not seen anyone suffering from this disease. This disease is a lie*.” Another stated that she does not understand how a disease kills people of the same age. She said: *“When an old man dies, they say it is not Ebola. How did the disease choose the young and not the old*?” In this group, few women believed in the existence of the EVD. The participants talked in detail about the practices of the *response*, which are powered by rumors about the vaccine, treatment, and types of patients sought. Participants mentioned more than 10 rumors about the Ebola virus, vaccines, treatments, and the SDB.

In a discussion in Butakuka, in a district of Beni, a man stated that the EVD remained a mystery to him and that he did not understand it. He said that the disease had lasted longer than usual, as it usually did not exceed six months. In his opinion, it was another disease, not Ebola, and that it amazed him that people were still feeding on these notions. In the same group, another person shared: *“We could stay and talk about these issues until night. We are never going to finish talking about it. You’ll fill up that notebook you have, we won’t finish talking about it. We have too many questions to ask you. Everything you say about the Ebola is the same as malaria. The symptoms are the same: vomiting, diarrhea, high temperature. Where did the Ebola come from? Is it a disease that was sent? Is it a common disease*?” The discussions often ended with participants sharing their questions. It was clear from these discussions that some people questioned the existence of the EVD, but they still wanted more information about it. We also found that participants had considerable information. For most of the questions asked, there were usually one or two participants who knew the answer. All participants were interested in the topics discussed. Participants were provided the opportunity to ask questions. In some cases, such as in Mbogu, in the Rwampara health zone, they asked more questions than were raised in the discussion. All the participants in this discussion agreed on the existence of the EVD, but they all knew someone who did not believe in it—some who thought it was an imaginary disease, and others who thought that it was an invented disease.

It became apparent that participants could ask several types of questions in a discussion. They could also ask questions that they knew the answer to or questions that some of them knew the answer to. Some of the questions were meant to hide rumors or to obtain information about rumors. Some also revealed suspicions, for example, that some of the smoke would be intended to contaminate and that difference in color was not accidental. The enquiries also revealed some of the concerns community members had.

We can exchange with the community members on topics that come from us. However, they can come up with all kinds of questions, including some about mechanics, related to the engines of vehicles or the smokes that come out of the pipes. If there are no answers to their questions, it can lead to more rumors and gossip about the people who are responsible for the *response*. During this research, we heard from members of *response* teams that they are not only observed, but also judged through their words. Consequently, any interaction with community members becomes a challenge.

In a group of young people from Mbogu, none of the participants questioned the existence of the EVD, but they all knew people who did not believe in its existence. This suggests that there are many people in this community who do not believe in the reality of the EVD, and that the denial of the Ebola virus’ existence comes from various people from all social strata. During the interviews and in some FGDs, young people, irrespective of gender, were considered as the ones who least believed in the existence of the EVD.

Throughout the interviews, very few traditional therapists questioned the existence of the Ebola virus. Besides village chiefs, avenue chiefs, and street chiefs, there are also traditional therapists who believe in the existence of the disease and play an important role in the response, as discussed below. However, at the time of this research, there were people among these groups who denied the existence of the EVD. Most of the individuals who denied the EVD’s existence were among general community members. Although village chiefs and community leaders have been educated on the EVD, we met one who refused to believe in the existence of the disease and therefore made no effort to contribute to the response. Social diversity is evident in the denial of EVD, as there are deniers in all strata. Community leaders were identified as the least likely group to be among those who deny the existence of the disease. This is possibly due to rumors about the EVD. Resistance and reluctance are encouraged by denial and rumors.

**Figure 1:**
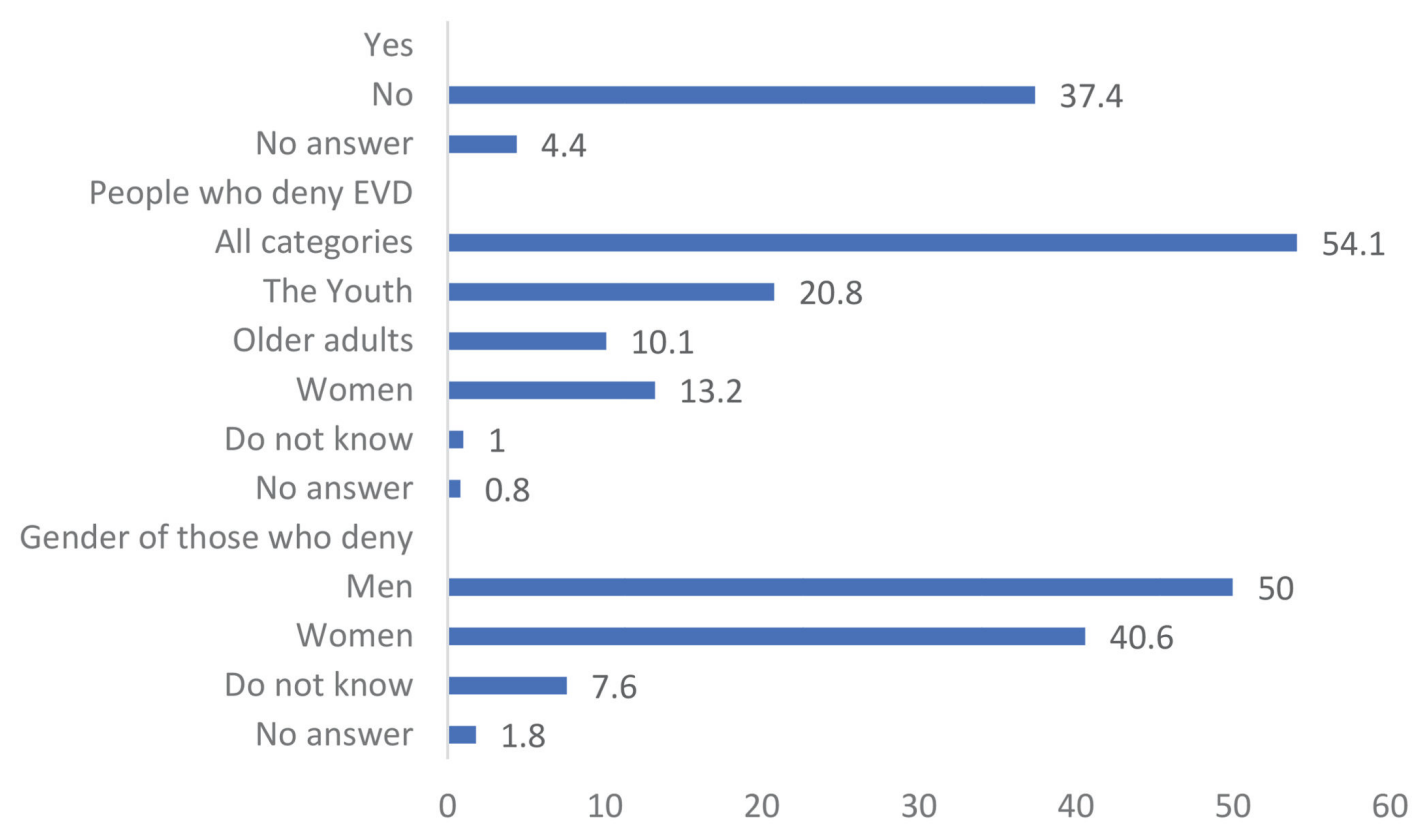
Distribution of respondents by knowledge and the categorization of people who deny EVD existence

Data from the questionnaire survey further provided statistical information corresponding to the attitudes of the people. Figure 1 reveals that more than half of the people interviewed knew others who did not believe in the existence of the EVD. This is significant as it may indicate that they know many people who do not believe in the existence of the EVD. If we consider the people who knew others who did not believe in the existence of the EVD, the numbers indicate that more than half of them were young people, women, and older adults (54.1%). Those who answered negatively were more than those who answered positively. When we asked for more details about these people, it appeared that men more commonly disbelieved in the existence of the disease, as shown in the table below.

Overall, men were more likely to not believe in the existence of Ebola. Denial of the existence of the disease contrasts with its classification as a priority health issue in communities.

Rumors about the Ebola virus are more prominent in urban populations than rural populations. The difference is significant at 0.001 with an χ^2^ of 10.236. Moreover, the population that has a level of education equivalent to secondary school are most aware of the rumors concerning the EVD.

Those with secondary and graduate levels of education had heard more rumors about the Ebola virus than others. All six people with postgraduate levels of education had heard rumors about the Ebola virus. Those with primary education or no education were less aware of rumors concerning the Ebola virus. The difference is significant at 0.001. The χ^2^ is 37.775. The rumors heard overlap with those cited by the sample in the qualitative surveys.

The most widespread rumor is the denial of the EVD, heard by almost a third of the population. This is also evident in qualitative surveys. Next is the rumor that the Ebola virus is for financial gain, heard by over a quarter of those who had heard rumors about the disease. Among the other rumors are those about witchcraft, politics, massacre, and human sacrifice. Some rumors are about the ETC and others about treatment, while some rumors classify the Ebola virus as a disease that cannot be treated by modern medicine. In the IDIs and FGDs, rumors about revenge, conflict, and punishment were not present. However, they were mentioned as rumors in the province of Equateur. These rumors come from several sources.

A third (33.6%) of the rumors mainly originated from the inhabitants of the village or neighborhood. These rumors were stated to have initially been heard from a friend, neighbor, or relative, who heard it before at the market, outside the village or neighborhood. Social networks also played a role in spreading the rumors, and some people stated that they have learned about the rumors at the springs or other places where water is collected. Furthermore, the radio has also been stated as a medium through which rumors are spread. Even though the people who heard the rumors for the first time through social networks or the radio are not numerous, it is important to note that resources are allocated for these means of disseminating information. It is of importance and has become a necessity to know the messages they are passing on the media and to monitor them. Among the sources of rumors, a teacher and a healthcare worker have also been identified, who are usually among the most informed individuals. Notably, the places where rumors were heard for the first time varied significantly.

Generally, rumors were first heard in the village or neighborhood. This is also where most of the mentioned sources can be found: friends, relatives, and neighbors. However, over a quarter of the people first heard the rumors outside their village or neighborhood. As the population moves and travels around, they also have access to information on the EVD, including rumors spread in markets, springs, and churches. Previously, in this study, we mentioned the case of a religious person in his place of worship who denied the reality of the EVD. Moreover, if a healthcare worker and a teacher were sources of rumors, the rumors were most likely not spread in their place of work.

Rumors about the EVD are not new in the provinces of North Kivu and Ituri. This was evident from the interviewees’ responses, of whom 97.5% had heard the rumors after the beginning of the epidemic. Some individuals had however heard the rumors 2−3 years ago, and others between 4−10 years ago. Although the EVD hit the country a long time ago, it was with the outbreak of the epidemic in the provinces of North Kivu and Ituri that the people we met first heard rumors about the EVD. Moreover, the rumors are not only about the disease, but also about the vaccines, and ETCs.

## Discussion and Conclusion

The response to the epidemic is slowed down by two major obstacles: denial of the disease and various rumors. While the existence of the EVD is denied, rumors are spread about the epidemic; the means, strategies, and the mechanism for fighting the disease; the ETCs; and the vaccines. The disease is denied by diverse groups of people, including those who play an important role in their community. Political leaders, village chiefs, neighborhood chiefs, street chiefs, and avenue chiefs are among those who question the reality of the EVD and its very existence. They attribute the disease to the transgression of a taboo or a curse. Thus, they do not adhere to the strategies developed by the authorities to fight the epidemic as they claim the EVD does not exist. This situation does not make it easy for the response teams to work with the communities. While some community leaders have contributed to the fight against the Ebola virus, others have denied the existence of the epidemic.

From the exchanges with the leaders, several facts are worth highlighting:

### Denial of the Disease

The denial of the disease was present in the arguments of a village chief, who also made some incorrect statements. According to him, there was no new illness, and this situation has remained unchanged since his youth. He referred to the past and ancient habits to question the existence of the EVD. He shared that hand washing is part of the behavior of people who wash their whole body, not just their hands.

The denial of the existence of the EVD leads people to reject any intervention that addresses it as a pathology. Popular interpretations deny the biomedical discourse on the origin of the disease and consequently, on the EVD itself^8^. These popular explanations attribute the cause of the disease to the transgression of taboos, such as touching fetishes. Denying the EVD has encouraged reluctance, resistance to interventions to fight it, and the epidemic it causes. In Macenta, Guinea, the reluctance originated from the divergence between the biomedical discourse and popular interpretations of the EVD or its origin^8^. Denial has been and is evident in all strata: among the public, teachers, traders, healthcare personnel, and even certain doctors. Some high-ranking political authorities have also denied the existence of the disease, and stimulated reluctance, resistance, and refusal of interventions to battle the EVD.

### Weight of the Rumors

In the words of a village chief, rumors were very common, and influenced attitudes toward certain issues related to the EVD epidemic. The disease was said to be fabricated and that it targeted people of blood group O. Meanwhile, doctors who were exposed to the disease did not die, which corroborated the rumors about target selectivity. The Ebola epidemic was presented as a business. The village chief also spoke of the Ebola epidemic as a private matter.

### Information on the EVD and its Prevention

Information on the EVD and how to prevent it is provided by the response teams. It is thus important to know what is done with the information and what it leads to. A village chief added that providing information was not enough as it was more important to know whether the information shared was accepted. The exchange of information during awareness-raising sessions is therefore essential. One must interact with the community members to inform them and it should not be offered as something optional. Furthermore, it is important to know the reasons why information is accepted and why it is refused or even considered a lie.

### Information First, Vaccination Second

For a village chief, information on the vaccine must be provided before any act of vaccination. He continued saying that information is—for any kind of intervention, including immunization—a prerequisite, something indispensable before the intervention is implemented.

### Conditions for Success

Regarding handwashing, a village chief first talked about water supply and availability. If there are no conditions to allow for a change of behavior, it will not exist or will not be effective. The availability of potable water becomes a *sine qua non* condition for handwashing.

### Addressing Health Issues Other than Ebola

One village chief had a more systemic approach to health. He believed that all health problems deserve to be addressed to the extent of those related to the Ebola epidemic. In addition to EVD prevention, he also considered that actions to prevent malaria are an urgent health issue in his community.

### Disruption Caused by the Ebola Response Interventions

A village chief criticized the interference on funerals due to preventive measures and the practice of SDB. According to him, they affected traditions negatively.

### Community Resources for the Response

If the village chief was involved, he could make community resources available to the response. It is the case of *Kibuyi*, which is being used to inform the villagers, for example. Information on the disease has not always been communicated through interaction with the communities but often *via* reports by the media, which are usually full of representations and stereotypes of the affected communities and their cultures^9^. Media has focused on the limitations, impediments, backwardness, and ignorance of the practices, customs, and beliefs of the communities. Community resources for the response include, beyond human resources, the culture of the communities where we can find leverage to support the response^1^.

### Grounds for Resistance to Build the Response

It is important to understand the root causes of resistance to improve the response. From a village chief’s comments, it became clear that there are several reasons for resistance. Once these reasons are known, it is easier to develop strategies to deal with them, namely those related to risk communication and community involvement. Risk information has been a major concern and it is not free of trouble. Even Western media and health experts from Western countries have not been immune to interpretations of Ebola-based changes in representations of the cultures of communities in the affected countries. These media have focused on myths, risks, and the lack of appropriate knowledge in the communities^10^. It is not only important to shape what needs to be said in activities purposed to inform and raise awareness, it is equally essential to analyze the situation and identify the breeding grounds for resistance, reluctance, refusal, and violence against the response interventions. For instance, the reasons for this leader’s low involvement in the response are known, as is evident from his statements.

### Possibility of Changing an Attitude

Attitude changes lead to selective involvement in the Ebola response interventions. A village chief, for instance, was not against the activities carried out to fight the EVD epidemic. He even encouraged the population to listen to the response teams. He acted according to his responsibilities, while remaining neutral. He noticed that some people listen while others do not. Furthermore, some advice is followed, such as washing hands, not eating the animals that died of natural causes, and not going to funerals. However, he considered these measures as rupturing relationships between people. In this regard, he also had an opinion on the new habits that have arisen due to the Ebola epidemic. His attitude was dynamic. For example, as a village chief, he welcomed the members of the response teams and allowed them to do their work, even though he was certain that not everyone would listen to them.

In addition to community leaders, other members of the community have also denied the existence of the disease. These include community members, the public, teachers, and even healthcare workers. There were even healthcare workers who were infected by the disease because they did not believe in its existence and therefore did not take the necessary precautionary measures during their professional practice. Throughout the interviews and FGDs, participants stated that they were aware of other community members who did not believe in the disease. The same was true for those surveyed by questionnaire. Denial of the disease has been a common feature of the response to the Ebola epidemic in the provinces of North Kivu and Ituri in the DRC. Denial of the existence of the disease by community members has been one of the biggest obstacles to the response. Furthermore, the denial of the disease by community leaders has been a hindrance to the implementation of the response activities in the areas for which they are responsible. Denial of the disease has led to reluctance, indifference, and refusal to participate in response activities. It has also led to acts of violence against members of the response teams, particularly the staff responsible for communication and community involvement, vaccination and, above all, SDB. The denial of the EVD was the most common phenomenon, common to men, women, older adults, and the young. Acts of violence against the *response* teams were usually committed by young people.

The second obstacle to the response was various rumors. Some of the rumors were about denying the existence of the reality of the EVD, as well as that of the epidemic. There were also rumors about the ETCs and the hospital, about the vaccine, and about SDB. Over 50 different rumors about the Ebola virus and the responses to these rumors were collected from clerics, traditional therapists, men, and women in FGDs, including rumors from healthcare personnel. Some rumors presented the EVD as an invention, a created disease, while others attributed it to witchcraft or an evil spell. The EVD is also seen as a scheme, a white man’s business, a business to make profits, to exterminate populations, and to destabilize elections. There are even rumors claiming there are no sick people.

Of the 40 rumors about the vaccine, many portrayed it as a tool to reduce the life expectancy of the population, to prevent reproduction, to cause diseases, or to reduce people’s intelligence. There were rumors about the existence of real vaccines, where it is believed that these are administered to healthcare workers and fake ones are used for the general population. The ETC and the hospital are also at the center of the rumors, with over 30 stories collected. The ETC is presented as a place where genitals and other organs are removed, people practice occult rituals, and death is induced. These rumors are not likely to encourage the acceptance of the ETC as a place for treatment. They are at the root of the reluctance to send a confirmed patient to a treatment center or the reason a patient flees from the treatment center after being admitted.

While there are fewer rumors about SDB, those that do exist are not conducive to public acceptance of the practice. They also revolve around the removal of organs and the interpretation of figures that would be on some body bags, some of which are considered as predictive of the number of deaths that will occur in the same family.

In conclusion, the EVD response has done much to address the first barrier, which is the denial of the disease and the existence of the epidemic. For example, it has been able to use information to involve community members who were initially hostile. The response teams have, however, done less to address the second obstacle. Knowledge of these rumors by the response authorities can be an asset in identifying strategies and means to deal with them.

## Figures and Tables

**Figure 2:**
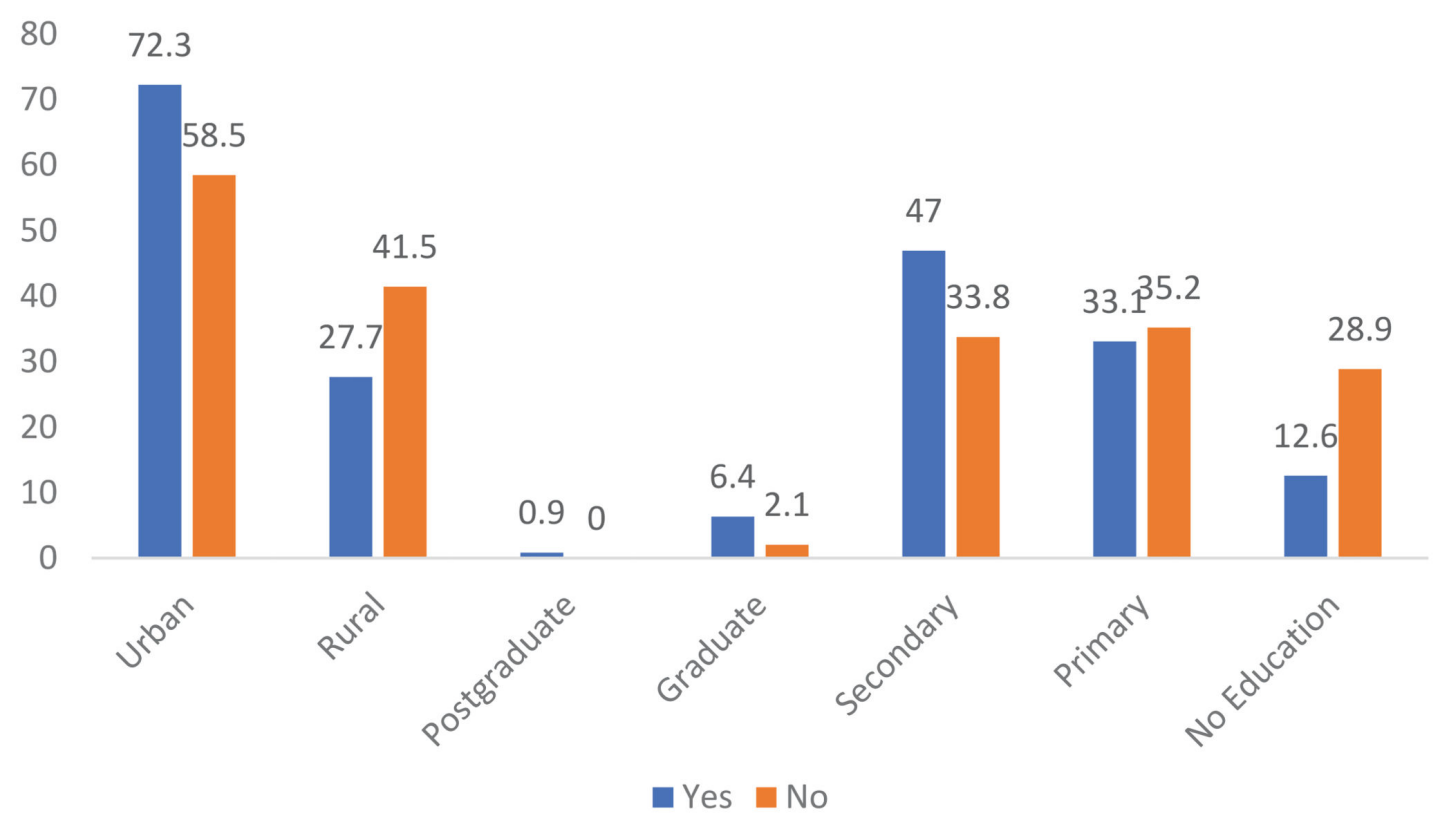
Distribution of respondents on agreement with rumors on EVD by locality and level of education

**Figure 3:**
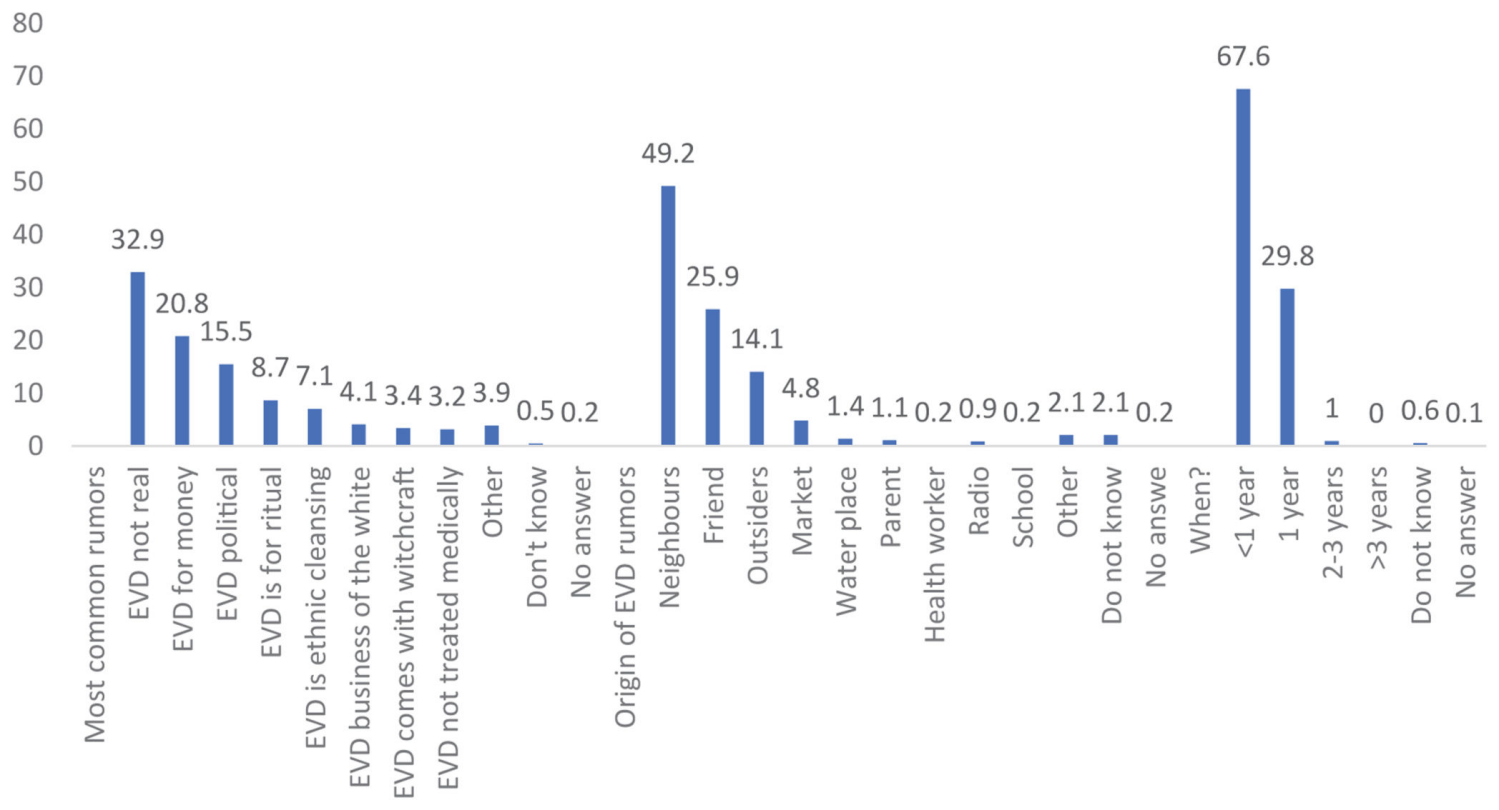
Distribution of respondents on perceived EVD rumors that are most common, their origins, and when they were first heard

**Table 1:** Distribution of the participants in the in-depth interviews (IDIs) and focus group discussions (FGDs) by provinces

Target	North Kivu Province				Ituri Province			
	Butembo		Beni		Mbuti		Bunia	
	IDI	FGD	IDI	FGD	IDI	FGD	IDI	FGD
Pillar leads	All		All		All		All	
Pillar members	2/pillar		2/pillar		2/pillar		2/pillar	
Community leaders^1^	≥2/community		≥2/community		≥2/community		≥2/community	
Leader of survivor group	≥2/community		≥2/community		≥2/community		≥2/community	
Community adult males		≥2 groups		≥2 groups		≥2 groups		≥2 groups
Community adult females		≥2 groups		≥2 groups		≥2 groups		≥2 groups
Community male youth		≥2 groups		≥2 groups		≥2 groups		≥2 groups
Community female youth		≥2 groups		≥2 groups		≥2 groups		≥2 groups
Survivors		≥2 groups		≥2 groups		≥2 groups		≥2 groups

1 community leaders here refer to political, opinion, traditional and religious leaders

## Data Availability

The data that support the findings of this study are not publicly available as they contain information that could compromise the privacy of the research participants. The data are available from the corresponding author (Joseph Okeibunor) upon reasonable request.
